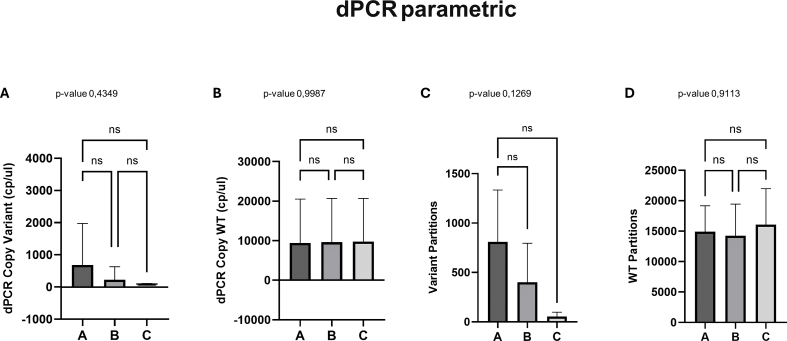# Harmonization trial on *ESR1* testing strategies in ER+/HER2- breast cancer patients: an Italian experience

**DOI:** 10.1016/j.jlb.2025.100314

**Published:** 2025-07-28

**Authors:** Francesco Pepe, Gianluca Russo, Konstantinos Venetis, Claudia Scimone, Lucia Palumbo, Mariantonia Nacchio, Domenica di Giovanni, Claudia Sarracino, Ilaria Tomaiuolo, Elisabetta Zulato, Matteo Fassan, Daniela Righi, Giuseppe Perrone, Dario de Biase, Gabriele Casati, Fabio Pagni, Lucia Gullotti, Antonio Rizzo, Alessandro Perez, Antonio Russo, Katia Zavaglia, Gianluca Gragnano, Domenico Cozzolino, Sabrina Alfano, Cristian Scatena, Sara D’andrea, Monica Schiappacassi, Giuseppina Roscigno, Alessia Paganotti, Renzo Boldorini, Francesco Esposito, Pierlorenzo Pallante, Daniela Furlan, Marta Sbaraglia, Angelo Paolo Dei Tos, Giancarlo Troncone, Nicola Fusco, Umberto Malapelle

**Affiliations:** a Department of Public Health, Federico II University of Naples, Via S. Pansini, 5, 80131, Naples, Italy; b Division of Pathology, European Institute of Oncology, IRCCS, Milan, Italy; c Department of Pathology, Azienda Ospedale-Università Padova, Padua, Italy; d Department of Medicine - DIMED, University of Padua, Padova, Veneto, Italy; e Veneto Institute of Oncology - IOV - IRCCS, Padova, Italy; f Anatomical Pathology Operative Research Unit, Fondazione Policlinico Universitario Campus Bio-Medico, Via Alvaro del Portillo, 200, 00128, Roma, Italy; g Research Unit of Anatomical Pathology, Department of Medicine and Surgery, Università Campus Bio-Medico di Roma, Via Alvaro del Portillo, 21, 00128, Roma, Italy; h Solid Tumor Molecular Pathology Laboratory, IRCCS Azienda Ospedaliero-Universitaria di Bologna, Bologna, Italy; i Department of Medicine and Surgery, Pathology, IRCCS Fondazione San Gerardo Dei Tintori, University of Milano-Bicocca, Milan, Italy; j Division of Pathology, Humanitas Istituto Clinico Catanese, 95045, Catania, Italy; k Section of Medical Oncology, Department of Precision Medicine in Medical, Surgical and Critical Care (MEPRECC), University of Palermo, 90127, Palermo, Italy; l Division of Molecular Genetics, Department of Laboratory Medicine, Pisa University Hospital, Pisa, Italy; m Division of Pathology, Department of Translational Research and New Technologies in Medicine and Surgery, University of Pisa, Pisa, Italy; n Department of Oncology, Pisa University Hospital, Pisa, Italy; o Molecular Oncology Unit, Centro di Riferimento Oncologico di Aviano (CRO) IRCCS, National Cancer Institute, Aviano, PN, Italy; p Department of Biology, Complesso Universitario Monte Sant'Angelo, University of Naples Federico II, Via Cintia 4, 80126, Naples, Italy; q Department of Pathology, 'Maggiore della Carità' Hospital, Novara, Italy; r Department of Health Sciences, Universitá degli Studi del Piemonte Orientale "A. Avogadro", Novara, Italy; s Institute of Endotypes in Oncology, Metabolism and Immunology (IEOMI) "G. Salvatore", National Research Council (CNR), Naples, Italy; t Department of Molecular Medicine and Medical Biotechnology (DMMBM), University of Naples "Federico II", Naples, Italy; u Unit of Pathology, Department of Medicine and Technological Innovation, University of Insubria, Varese, Italy; v Department of Integrated Diagnostics, Azienda Ospedale-Università Padova, Department of Medicine, University of Padua, Italy; w Department of Oncology and Hemato-Oncology, University of Milan, Milan, Italy

**Keywords:** Breast, Molecular pathology, Tumour biomarker

## Abstract

**Aims:**

To date, *ESR1* activating mutations acts as key player to clinically stratify estrogen receptor (ER)+/HER2-advanced breast cancer (BC) patients eligible to novel new generation oral Selective Estrogen Receptor Degraders (SERD) relapsing after first line aromatase inhibitors. Liquid biopsy represents the most useful biological source to detect *ESR1* activating mutations in clinical setting, but the lack of standardized pre-analytical and analytical procedures drastically impacts on detection rate of *ESR1* mutations in diagnostic specimens. Here, we sought to harmonize technical procedures comparing technical performance of diagnostically available testing strategies on a series of three reference specimens (sample A, B, C) harboring *ESR1* p.D538G mutation at different mutant allele fraction (MAF) (5.0 %, 1.0 %, 0.5 %) shared with n = 10 Italian referral institutions.

**Methods:**

A total of 10 μl of gDNA from each reference sample built to mimic clinically detectable *ESR1* molecular alteration (5.0 %, 1.0 %, 0.5 % VAF) was shipped by coordinator institution to each participating group to test p.D538G *ESR1* alteration leveraging own routinely available testing strategy. Artificial reference sample was previously validated by the University of Naples Federico II before arranging the shipment.

**Results:**

*ESR1* exon 10 p.(D538G) hotspot mutation was successfully identified in 90.0 % of samples A, B whereas 8 out of 10 (80.0 %) participating institutions detected sample referenced alteration in sample C. No statistically significant variations were observed between dPCR and NGS based workflows in terms of detectability rate on standard reference samples. A single participating institution (ID#5) failed to detect p.(D538G) *ESR1* alteration but supervised procedures by coordinator institution enabled to detect referenced mutation in engineered reference samples set adopting an orthogonal technology (dPCR). In addition, NGS and dPCR platforms displayed a similar technical performance in detecting *ESR1* across samples A-C.

**Conclusions:**

NGS and dPCR systems may be considered valid technical solutions to target low frequency *ESR1* alterations in diagnostic routine samples. Harmonized ring trials are key weapons to standardize analytical and post-analytical procedures optimizing clinical stratification of BC patients

## 1 Introduction

Nowadays, tailored options have shifted the clinical paradigm for breast cancer patients (BC). Firstly, Phosphatidylinositol-4,5-Bisphosphate 3-Kinase Catalytic Subunit Alpha (*PIK3CA*) was approved by international societies for the clinical stratification of BC patients expressing specific receptor profile [hormone receptor (HR)+/HER2-] [1,2]. Recently, it has been established that estrogen receptor (ER) alpha (*ESR1*) activating mutations play a pivotal role in driving tumor progression in (HR)+/HER2- BC patients after first line of endocrine therapy (ET) [3,4]. Of note, *ESR1* dependent resistant mechanisms occur in 40.0 % of metastatic (HR)+/HER2- BC patients relapsing from aromatase inhibitor (AI) plus endocrine regimen [5]. In this scenario, liquid biopsy emerged as the most promising biological source for recovering nucleic acids to test *ESR1* in the diagnostic routine practice of HR+/HER2- BC patients. The clinical rationale derives from the clinical benefit guided by *ESR1* mutations in (HR)+/HER2- BC patients treated with selective ER degraders (SERDs) and ER modulators (SERMs) compared with standard of care endocrine monotherapy [6,7]. Blood based *ESR1* (*bESR1*) activating alterations were investigated in clinical trials where *bESR1* mutant BC patients were elected to ER inhibitors after molecular profiling of liquid biopsy samples [8,9]. Phase III EMERALD trial (NCT03778931) demonstrated improving rate of progression-free survival (PFS), in randomized ER+/HER2–advanced BC patients harboring *ESR1* activating mutations under elacestrant (oral SERD) in comparison with standard of care (SOC) [2,10]. In addition, the phase III PADA-1 trial (NCT03079011) prospectively investigated the clinical relevance of switching therapy in bESR1mut BC patients after first-line AI randomizing patients to fulvestrant or maintaining the same therapeutical scheme in accordance with *bESR1* molecular assessment [11]. Phase 3trial SERENA-6 (NCT04964934) confirmed clinical efficacy of switching from AI to novel generation oral SERD (Camizestrant) detecting *ESR1* mutations in liquid biopsy samples before clinical progression from standard of care [12]. Remarkably, distinct technological approaches were adopted in the aforementioned trials: the former was built on a comprehensive next generation sequencing (NGS) assay (Guardant360® CDx, GuardantHealth, CA, USA) able to simultaneously detect clinically informative molecular alterations across 55 cancer related genes from liquid biopsy specimens, including *ESR1* activating mutations (310–547 codons); the latter on customized digital droplet PCR (ddPCR) assays to analyze four hotspot mutations covering 90.0 % of most frequent *ESR1* actionable alterations [2,11]. Preclinical studies identified more than n = 60 hotspot molecular alterations in ligand-binding domain (LBD) impacting on the constitutive activation of signaling pathway. Given the heterogeneous distribution of *ESR1* activating mutations and the impressive number of predictive biomarkers approved in clinical practice NGS platforms are recommended but a not negligible percentage of institutions addicted to molecular tests routinely adopt single-plex platforms [8,13,14].

Not surprisingly, each testing strategy harbors distinct technical parameters in terms of accuracy rate, reference range and turn-around time (TAT) impacting on standardization of analytical procedures [15,16]. On this basis, the lack of standardized processes could decrease the number of patients eligible for target therapy. To fill this gap, harmonized handling protocols increasing the detection rate of *ESR1* hotspot mutations in BC patients are required. Hence, we designed a multicenter trial involving n = 10 highly experienced Italian Institutions in molecular testing to analyze a *ESR1* activating mutation on a customized reference sample at different variant allele fraction (VAF). Then, concordance rates were evaluated by comparing the molecular results shared by each participating institution.

## 2 Methods

### 2.1 Study design

A series of three standard reference samples (sample A, B, C) harboring *ESR1* p.D538G (NM_001291230.2) activating mutation at scalable VAF (5.0 %, 1.0 %, 0.5 %) were shared by the coordinator center with nationwide institutions involved in molecular tests. This series was able to cover mostly recurrent VAF levels in real-life molecular diagnosis of *ESR1* positive ER+/HER2 BC patients [17]. Artificial control was built engineering pRP-CMV-hESR-WT plasmid with p.D538G *ESR1* mutation. Before shipping standard reference samples to participating institutions, coordinator institution (University of Naples Federico II) internally validated sample set adopting both NGS platform and dPCR assay, in accordance with clinical trials of elacestrant [10]. Briefly, fragmentation rate of nucleic acids from pRP-CMV-hESR engineered samples was also evaluated by microfluidic platform validating quality/quantity of gDNA before molecular analysis on the most recommended platforms by international societies [18] After internal validation, a sample set grouping three *ESR1* p.D538G engineering samples (5.0 %; 1.0 %; 0.5 % VAF) was sent to all participating institutions (n = 10). Each institution analyzed standard reference samples following its own routinely implemented diagnostic workflow. Finally, technical and molecular records on *ESR1* molecular analysis were annotated in dedicated form and sent to the coordinator institution (Fig. 1). Biological material was managed under the authorization of Department of Public Health at the University of Naples Federico II, Naples.

**Fig. 1:**
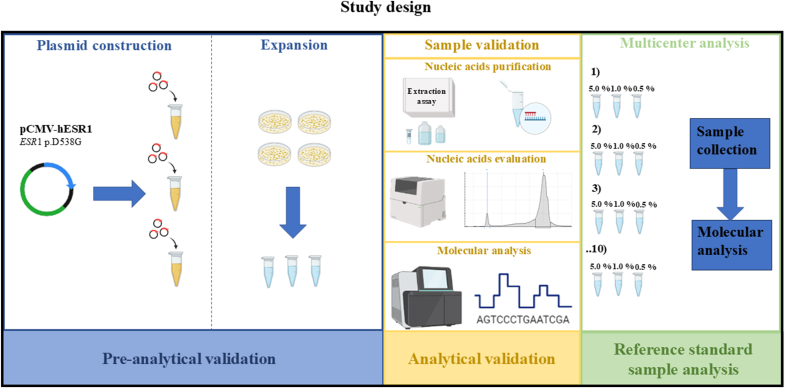
Schematic representation of study design. Briefly, after internal validation of standard reference sample at University of Naples Federico II, a set of standard reference samples built to mimic *ESR1* 5.0 %, 1.0 % and 0.5 % p.D538G mutations were shared with n = 10 referral institutions after internal validation. Here, own diagnostic workflow (from Nucleic Acid extraction to molecular data interpretation) was approached. Data were shared with coordinator center. *Abbreviations:* CMV (cytomegalovirus promoter), ESR1 (Estrogen receptor-1).

### 2.2 Standard sample generation and validation

The plasmid vectors expressing the CDS contained in the hESR1 ORF [NM_001291230.2] under the control of the CMV promoter were custom-designed and generated at the VectorBuilder Inc. company (Chicago, IL, USA). The plasmid vectors used as positive controls express the CDS contained in the ORF NM_001291230.2 corresponding to hESR1-WT (pRP-CMV > hESR1-WT) and the corresponding CDS in which the GAC > GGC (pRP-CMV > hESR1-D538G). Each engineered sample was expanded following standardized procedures for bacterial clones [19]. Then, pRP-CMV > hESR1-D538G (100.0 % VAF) was mixed with wild type *ESR1* samples (pRP-CMV-hESRWT) to arrange a longitudinal series of reference specimens covering seven experimental points ranging from 100.0 % to 0.5 % of VAF [10,11]. internally confirmed on a dPCR system (Supplementary Fig. 1).

### 2.3 Nucleic acids qualification

Focusing on three experimental points (pRP-CMV > hESR1-D538G 5.0.-0.5 % VAF), gDNA was evaluated on 4200 TapeStation system (Agilent Technologies, Santa Clara, California, USA), a fully automatized microfluidic platform, in terms of nucleic acids amount (ng/μl) and fragmentation index [2]. As regards, 1 μl of genomic DNA was manually combined with 10 μl of proprietary buffer and automatically loaded into a Genomic ScreenTape cartridge (Agilent Technologies, Santa Clara, California, USA). Then, nucleic acids were analyzed on 4200 TapeStation system (Agilent Technologies, Santa Clara, California, USA), in accordance with manufacturer protocol. Finally, DNA integrity number (DIN), scored between 0 (high-fragmented profile) and 10 (low-fragmented profile), was interpreted by using the TapeStation 4200 analysis software (Agilent Technologies, Santa Clara, California, USA).

### 2.4 Molecular analysis

QuantStudio Absolute Q Digital PCR System (Thermo Fisher Scientific, Waltham, Massachusetts, USA) was used in accordance with manufacturer instructions to test reference standard samples. Briefly, 1 μl of engineered gDNA was combined with Absolute Q™ DNA Digital PCR Master Mix 5X (Thermo Fisher Scientific, Waltham, Massachusetts, USA) and Custom SNP Genotyping product dPCR Assay (Thermo Fisher Scientifics, Waltham, Massachusetts, USA) covering n = 5 most common *ESR1* activating mutations (exon 10 p.L536R, p.Y537C, p.Y537N, p.Y537S, p.D538G). Each well was complemented with QuantStudio™ Absolute Q™ Isolation Buffer (Thermo Fisher Scientifics, Waltham, Massachusetts, USA) supporting DNA fragments’ diffusion across wells on QuantStudio™ Absolute Q™ MAP16 Plate (Thermo Fisher Scientifics, Waltham, Massachusetts, USA). Thermal profile, partitioning procedure and signal detection were assessed on proprietary analysis software QuantStudio Absolute Q Digital PCR System Software (Thermo Fisher Scientifics, Waltham, Massachusetts, USA) maintaining standardized technical parameters. Positive signaling threshold was manually set at 5000 RFU for inspecting both mutant and wild-type signal. The total amount of copies/μl and matched MAF level were calculated by fractioning the number of positive partitions on the total valid partitions (based on Poisson distribution) in each well.

The fully integrated Genexus™ platform (Thermo Fisher Scientific, Waltham, Massachusetts, USA) was adopted on validation series of standard reference samples. As regards, libraries were generated adopting the Oncomine Precision Assay (OPA) panel, able to cover the most clinically informative molecular alterations across n = 50 reference genes (including *ESR1* ligand binding domain), in accordance with the manufacturer’s instructions [5]. Each sample was manually diluted and immediately dispensed on a 96-well plate equipping technical platform. Sequencing was approached on GX5TM chip following manufacturer procedures. Briefly, data analysis was carried out by variant calling plug-in based on Oncomine Knowledgebase Reporter Software (Oncomine Reporter 5.0) integrated in Genexus software (v.1.0). In addition, BAM files were also visually inspected targeting *ESR1* ligand binding domain (LBD) mutations on Golden Helix Genome Browser v.2.0.7 (Bozeman, MT, USA). Variants showing coverage ≥500X and mutated reads >10X were annotated.

### 2.5 Statistical analysis

Continuous data are presented as mean ± standard deviation. A statistical significance was set at a two-tailed p-value of 0.05. All statistical analyses, including Student’s t-test for comparisons between two groups and one-way or two-way ANOVA followed by Tukey’s post hoc test for multiple group comparisons, were conducted using Prism GraphPad software, version 10.0 for Windows (GraphPad Software, Boston, MA, USA; www.graphpad.com).

## 3 Results

### 3.1 Standard sample generation and validation

Overall, A, B, C samples highlighted 4.1 ng/μl, 4.3 ng/μl and 4.2 ng/μl, respectively. A DIN of 7.7, 7.4 and 7.5 were also inspected demonstrating high quality of DNA profile (Fig. 2). The sample series was successfully evaluated in duplicate adopting both NGS and dPCR systems (Supplementary Table 1). Particularly, NGS quality checks were satisfied for each engineered sample: a median number of 3168695.5, 1133152.0 and 1850359.0 total reads; mean read length of 106.0, 103.0 and 104.0; number of mapped reads of 3132220.0, 1112778.5 and 1823408.0, a percent reads on target of 72.2 %, 73.4 % and 75.0 %, a mean depth of 10292.0, 3612.5 and 6132.0 were inspected in samples A, B and C, respectively. Moreover, dPCR system reached a total of 9483.2, 69.8 and 20.9 copies/μl in sample A, B, and C, respectively, manually filtering threshold at 5000 RFU. Of note, NGS (median 4.4 %, 1.1 %, 0.3 %) and dPCR (4.8 %, 1.0 %, 0.6 %) confirmed VAF levels of *ESR1* p.D538G activating mutation in reference standard sample set from two independent experimental set up (Table 1) (Supplementary Table 1)

**Fig. 2:**
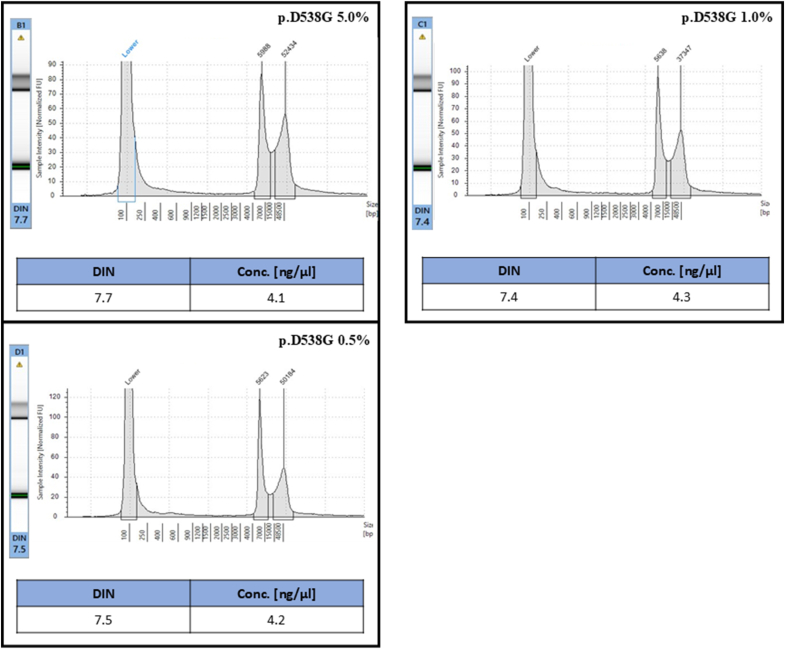
Internal validation - nucleic acids quantifications and qualifications by using TapeStation4200 platform (Agilent, Santa Clara, CA, USA) on standard reference samples A, B, and C.

**Table 1:** Molecular results from internal validation by coordinator institution on reference standard samples A-C adopting NGS and dPCR sysems (Thermofisher Scientifics).

Technology	Platform	Assay	Sample A (5.0 %)	Sample B (1.0 %)	Sample C (0.5 %)
NGS	IonTorrent Genexus Integrated Sequencer	Oncomine™ Precision Assay GX	4.4 %	0.8 %	0.3 %
			4.4 %	1.4 %	0.3 %
dPCR	QuantStudio Absolute Q Digital PCR	Custom Assay	4.2 %	1.0 %	0.5 %
			5.3 %	1.0 %	0.6 %

### 3.2 Standard sample analysis

Overall, a sample set including aliquots (15 μl) from A-C engineered plasmids was successfully shared by coordinator institution with n = 10 participating laboratories. Technical data and molecular results were listed in a dedicated repository file and sent to coordinator institution within 60 working days. As suggested by experimental design, participating institutions adopted their own diagnostically available testing strategy to evaluate *ESR1* activating mutations in standard reference samples. Particularly, 6 out of 10 (60.0 %) and 4 out of 10 (40.0 %) institutions adopted NGS and dPCR systems, respectively. Among NGS group, amplicon-based, and hybridization-based platforms were used in n = 3 (50.0 %) and n = 3 (50.0 %), respectively, to detect *ESR1* p.DS538G hotspot mutation. Interestingly, a custom hybridization NGS assay was used in a single instance (ID#9). Given 4 out of 10 (40.0 %) institutions selected dPCR approach, a digital droplet PCR system (ddPCR) was used in 2 out of 4 (50.0 %) institutions, remaining cases (50.0 %) selected plate-based dPCR system for genotyping the *ESR1* most common hotspot mutations (n = 5) (Table 2).

**Table 2:** Technical approaches of each participating institutions evaluating p.D538G mutations in standard reference samples A-C.

Centre	Technology	Platform	Assay
**#1**	ddPCR	QX 200 BIO-RAD	ddPCR Supermix for Probes
**#2**	NGS	IonTorrent Genexus Integrated Sequencer	Oncomine™ Precision Assay GX
**#3**	dPCR	QuantStudio Absolute Q Digital PCR	Custom Assay
**#4**	NGS	IonTorrent Genexus Integrated Sequencer	Oncomine™ Precision Assay GX
**#5**	NGS	Illumina Miseq	Amoy HRR NGS panel
**#6**	ddPCR	QX 200 BIO-RAD	ddPCR Supermix for Probes
**#7**	NGS	Illumina Miseq	Amoy HRR NGS panel
**#8**	NGS	IonTorrent Genexus Integrated Sequencer	Oncomine™ Precision Assay GX
**#9**	NGS	Illumina Miseq	Custom Assay
**#10**	dPCR	QuantStudio Absolute Q Digital PCR	Custom Assay

### 3.3 Data analysis

Overall, standard reference samples were successfully analyzed in all instances (see Table 3a). Particularly, *ESR1* p.D538G hotspot mutation was detected in 90.0 % of samples A, B whereas was identified in 8 out of 10 (80.0 %) sample C by participating institutions. Moreover, dPCR and NGS platforms detected p.D538G *ESR1* mutation in 4 out of 4 (100 %) and 5 out of 6 (83.3 %); 4 out of 4 (100 %) and 5 out of 6 (83.3 %); and 4 out of 4(100 %) and 4 out of 6 (67.0 %) A, B, C samples, respectively. NGS-based testing strategies showed a median number of 10608723.7 total reads (ranging from 1845577.0 to 25284921.0), of 10497515.8 mapped reads (ranging from 1822160.0 to 24924523.0), a median read length of 95.9 (ranging from 44.0 to 110.0), a percentage reads on target of 83.2 % (ranging from 71.2 % to 99.6 %) an average mean depth of 26011.3 (ranging from 2916.0 to 81069.0). No significant variations in terms of NGS technical parameters were observed among longitudinal samples (p-value >0.05) (Supplementary Fig. 2). In addition, dPCR platforms identified a median of 683.9 cp/μl, (ranging from 0.2 to 2992.0), 228.7 cp/μl (ranging from 0.8 to 947.0) and 40. 3 cp/μl (ranging from 0.3 to 164.0) in A, B, and C samples (Table 3 A-B). Similarly, no statistically significant variations were identified comparing dPCR technical parameters across longitudinal samples (p-value >0.05) (Supplementary Fig. 3). Among institutions adopting NGS systems, amplicon and hybridization-based approaches demonstrated a comparable technical performance in detecting *ESR1* p.D538G hotspot mutation in standard reference samples (3/3 amplicon-based and 2/3 hybridization-based strategies). Notably, a single participating institution (ID#5) failed to successfully detect p.D538G *ESR1* alteration in shared samples. Under the supervision of coordinator institution, molecular analysis of standard reference samples was carried out. Particularly, institution ID#5 switching to orthogonal technology (dPCR), was able to successfully identify referenced mutations in engineered samples set (Table 4). Remarkably, the trained program achieved a detection rate of 100.0 % and 90.0 % for standard reference samples A-B and C (9 out of 10), respectively, involving all participating institutions. Finally, a median VAF level of 4.8 (ranging from 0.58 % to 20.0 %), 1.6 % (ranging from 0.3 % to 4.0 %) and 0.5 % (ranging from 0.1 % to 2.5 %) were identified. In addition, NGS and dPCR systems highlighted no significant variations in terms of VAF measurement across samples A-C: 2.3 % (ranging from 0.6 % to 4.5 %) and 7.2 % (from 1.6 to 20.0 %); 0.9 % (from 0.3 % to 2.2 %) and 2.2 % (from 0.8 % to 4.0 %); 0.3 % (from 0.1 to 0.5 %) and 0.7 % (from 0.1 to 0.2.5). (p-value >0.05) (Table 4)

**Table 3a:** Technical parameters from NGS analysis performed by participant institutions on samples #A, #B, #C.

Centre ID/Sample		Total reads	Mapped reads	Mean depth	Uniformity of coverage	Mean read lenght	Percent reads on target
**#2**	**A**	2446707	2410532	7921	1.4 %	102	75.1 %
	**B**	1845577	1822160	5945	1.4 %	103	74.0 %
	**C**	2062012	2029117	6689	1.4 %	103	74.7 %
**#4**	**A**	25284921	24924523	81069	1.5 %	109	71.2 %
	**B**	20701400	20500036	67102	1.5 %	110	71.4 %
	**C**	20911216	20700872	67638	1.5 %	110	71.6 %
**#7**	**A**	17138288	17074876	6843	0.8 %	44	99.6 %
	**B**	7343666	7301807	2916	0.8 %	57	99.4 %
	**C**	8334780	8298940	3321	0.8 %	49	99.6 %
**#8**	**A**	12791112	12604425	41144	1.4 %	107	72.3 %
	**B**	12913329	12734547	4181	1.4 %	108	72.1 %
	**C**	14394599	14227287	47741	1.4 %	109	73.2 %
**#9**	**A**	3981808	3941990	14639	NA	109	98.0 %
	**B**	4106384	4065320	15097	NA	109	98.0 %
	**C**	4875056	4826305	17923	NA	109	98.0 %

**Table 3b:** Technical parameters from dPCR analysis performed by participant institutions on samples #A, #B, #C.

Centre ID/Sample		Copy Variant (cp/ul)	Copy WT (cp/ul)	Variant Partitions	WT Partitions
**#1**	**A**	51.0	3190.0	668.0	14701.0
	**B**	26.7	3000.0	240.0	9866.0
	**C**	6.5	4700.0	104.0	18481.0
**#3**	**A**	0.2	5.9	NA	NA
	**B**	0.8	48.8	NA	NA
	**C**	0.3	94.7	NA	NA
**#5**	**A**	2992.0	20091.0	369.4	10740.0
	**B**	947.0	20336.0	110.0	12947.0
	**C**	164.0	20074.0	18.7	9324.0
**#6**	**A**	203.0	1015.0	NA	NA
	**B**	68.4	1753.8	NA	NA
	**C**	26.4	1056.0	NA	NA
**#10**	**A**	173.4	22839.0	1391.0	19269.0
	**B**	100.7	22923.0	851.0	19987.0
	**C**	4.1	22973.0	36.0	20418.0

**Table 4:** B. Molecular result of MAF measurement on standard reference samples engineered with p.D538G mutation and corrective procedures (∗) enabled successful detection of p.D538G mutations among institutions #3 and #5.

Centre	Sample A (5.0 %)	Sample B (1.0 %)	Sample C (0.5 %)
#1	1.6 %	0.9 %	0.1 %
#2	3.4 %	2.2 %	0.4 %
#3	3.8 %	1.6 %	0.3 %
#3∗	NA	0.7 %	0.3 %
#4	1.5 %	0.3 %	0.1 %
#5	Failed	Failed	Failed
#5∗	3.3 %	0.8 %	0.2 %
#6	20.0 %	3.9 %	2.5 %
#7	0.6 %	0.6 %	nd
#8	1.7 %	0.3 %	0.1 %
#9	4.5 %	1.0 %	0.5 %
#10	7.2 %	4.0 %	0.2 %

## 4 Discussion

**Fig. 3:**
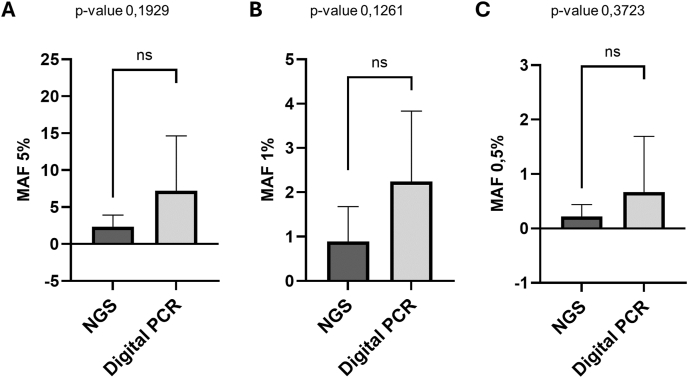
**MAF levels comparison among participating institutions**. Statistical analysis comparing VAF levels in standard reference samples (5.0 %, 1.0 %, 0.5 %) among the institutions (NGS or dPCR) was approached. Mean percentage ± SD of independent analysis (p-value >0.05) was calculated.

With the advent of the genomic era, the molecular landscape of advanced BC patients has been progressively improved revealing clinically significant predictive biomarkers able to shift the clinical paradigm for ER+/HER2- BC patients [20,21]. On this basis, challenging diagnostic specimens are controversial sources of nucleic acids to successfully perform genomic profile of clinically relevant predictive biomarkers due to scant diagnostic material [22,23]. Particularly, tissue biopsy may be affected by low quality nucleic acids depending on disharmonized preanalytical procedures [23]. Conversely, the low abundance of cfDNA in peripheral blood improves false negative rate in detecting druggable alterations in solid tumor patients [4], [24], [25]. On this basis, tissue and liquid biopsy samples became integrative tools in the clinical management of advanced BC patients identifying actionable alterations to guide therapeutic strategies [6], [26], [27]. Particularly, *ESR1* activating mutations should be tested to elect (10.13039/501100014832HR)+/HER2- BC patients, relapsing after first line therapy with aromatase inhibitors, to novel oral SERD [12,28]. It has been ascertained that *ESR1* actionable mutations are elicited as resistance mechanism of first line treatment supporting the central role of liquid biopsy to detect *ESR1* hotspot mutations [29]. In this scenario, liquid biopsy emerged as a dynamic, less invasive and technically consistent biological source of nucleic acids investigating *ESR1* mutations in first line resistant BC patients [30,31]. Behind the clinical implementation of liquid biopsy in clinical practice, several opening challenges drastically impact on *ESR1* molecular analysis [30,32]. Firstly, the lack of harmonized preanalytical procedures (inadequate collection time, disharmonized cfDNA preservation and purification protocols) affects *ESR1* detectability in diagnostic specimens. Secondly, analytical strategies are heterogeneous in terms of technical parameters (reference range, technical sensitivity) increasing inter variability among laboratories involved in *ESR1* molecular testing [12,33,34]. Here, we sought to evaluate technical performance of diagnostic routine workflows in n = 10 Italian referral institutions for molecular testing sharing an *ESR1* standard reference sample built to mimic VAF levels of real diagnostic samples. Being aligned with technical equipment of PADA-1 [11] and EMERALD [2] trials, both dPCR and NGS-based strategies were internally validated before the shipment of standard reference samples to participating institutions. Interestingly, dPCR systems require threshold set up because fluorescent signal also saturate empty wells in engineered samples. Conversely, NGS platforms were able to detect *ESR1* p.D538G hotspot mutation with VAF <1.0 % optimizing the bioinformatic pipeline to detect low-frequency molecular alterations. On this basis, coordinator institutions visually inspect NGS data to properly identify VAF <1.0 % *ESR1* alterations. These findings stress the need for harmonizing data interpretation procedures because molecular interpreting criteria, like the visual inspection of molecular records, could exclude ER+/HER2 BC patients from the best therapeutical strategy [33]. Additionally, molecular diagnosis of *ESR1* <1.0 % from diagnostic samples require highly sensitive technical platforms (dPCRs and NGS) that allow to identify low abundant alterations electing ER+/HER2- BC patients for oral SERD [34,35]. Opening challenges in the handling procedures of *ESR1* test on diagnostic routine samples significantly affect positivity rate suggesting that trained personnels act as key players to successfully administrate technical parts of molecular testing [3,6]. At the sight of this critical point, institution ID#2 inspected *ESR1* p.D538G molecular alteration in standard reference samples set under the supervision of coordinator institution supporting entire handling and data interpretation phase. In the same scenario, ID#5 implemented an NGS based testing strategy to target referenced alterations in artificial samples failing to yield molecular results due to hard technical procedures. Here, an orthogonal technology (dPCR) was adopted after an in-site training properly identifying *ESR1* p.D538G hotspot mutations in reference series. On this basis, diagnostic algorithm integrating orthogonal technologies should be implemented to adequately administrate diagnostic routine samples from BC patients ([7,8]). Sustaining this proof of concept, simultaneous analysis of standard reference samples was realized adopting a plate-based dPCR system and a BC tailored cfDNA based NGS panel in a single instance (ID#3). Interestingly, no statistically significant variations were identified comparing NGS and dPCR results on standard reference samples. (p-value >0.05) [36], [37], [38]. In addition, low technical intra-variability was observed in terms of technical parameters. In particular, VAF levels showed comparable results on sample A-C among participating institutions. (p-value >0.05) (Fig. 3). Our study shows some limitations, including the hard handling procedures to optimize technical analysis on engineered plasmid, the heterogeneous landscape of technical strategies to perform molecular tests across participating institutions and the lack of a more comprehensive standard reference series covering multiple *ESR1* mutations >5.0 % [[39], [40], [41]]. Additionally, manual set up of dPCR threshold and visual inspection of NGS raw data may impact on VAF detectability across participating institutions; in particular below 1.0 % [41]. On this basis, harmonization ring trials investigating *ESR1* testing procedures are insightful to check diagnostic workflow in diagnostic settings. Further investigations should be approached covering predictive biomarkers in ER+/HER2- BC patients also overviewing preanalytical handling procedures affecting molecular analysis of diagnostic samples.

## Patient consent for publication

Not applicable

## Author contributions

Conceptualization, Francesco Pepe, Gianluca Russo, Giancarlo Troncone and Umberto Malapelle.; methodology, all the authors; software, Francesco Pepe, Gianluca Russo; validation, all the authors; formal analysis, all the authors; data curation, Francesco Pepe, Gianluca Russo and Umberto Malapelle.; writing—original draft preparation, Francesco Pepe, Gianluca Russo; writing—review and editing, Giancarlo Troncone and Umberto Malapelle.; visualization all the authors; supervision, Giancarlo Troncone, and Umberto Malapelle.; project administration, Giancarlo Troncone and Umberto Malapelle All authors have read and agreed to the published version of the manuscript.

## Ethics approval

IRB approval is not required.

## Data availability statement

Data are available on request to the corresponding author. All data relevant to the study are included in the article or uploaded as supplementary information All data that are publicly available and used in the writing of this article in the text and the reference list.

## Funding

Monitoraggio ambientale, studio ed approfondimento della salute della popolazione residente in aree a rischio—In attuazione della D.G.R. Campanian.180/2019. POR Campania FESR 2014–2020 Progetto “Sviluppo di Approcci Terapeutici Innovativi per patologie Neoplastiche resistenti ai trattamenti—SATIN”. This work has been partly supported by a grant from the Italian Health Ministry’s research program (ID: NET-2016-02363853). National Center for Gene Therapy and Drugs based on RNA Technology MUR-CN3 CUP E63C22000940007 to DS.

## Declaration of competing interest

The authors declare the following financial interests/personal relationships which may be considered as potential competing interests: Francesco Pepe has received personal fees as speaker bureau from Menarini, 10.13039/100004337Roche, Thermofisher for work performed outside of the current study. Konstantinos Venetis has received honoraria for speaker bureau from Merck Sharp & Dohme (MSD), Roche, and AstraZeneca. "Cristian Scatena reports speaker bureau for Astra Zeneca, 10.13039/501100022274Daiichi Sankyo, Cerca Biotech, 10.13039/100005564Gilead, MSD; advisory role for Astra Zeneca, 10.13039/501100022274Daiichi Sankyo, 10.13039/100030841Exact Sciences, Menarini; consulting for Astra Zeneca, 10.13039/501100022274Daiichi Sankyo, 10.13039/100005564Gilead, Menarini, MSD; research grants from 10.13039/100005564Gilead. Giancarlo Troncone reports personal fees (as speaker bureau or advisor) from 10.13039/100004337Roche, 10.13039/100030732MSD, 10.13039/100004319Pfizer, Boehringer Ingelheim, Eli Lilly, BMS, GSK, Menarini, 10.13039/100004325AstraZeneca, 10.13039/100002429Amgen and 10.13039/100004326Bayer, unrelated to the current work. Nicola Fusco has received honoraria for consulting, advisory role, speaker bureau, travel, and/or research grants from 10.13039/100009947Merck Sharp & Dohme (10.13039/100030732MSD), 10.13039/100004334Merck, 10.13039/100004336Novartis, 10.13039/100004325AstraZeneca, 10.13039/100004337Roche, 10.13039/501100014337Menarini Group, 10.13039/501100022274Daiichi Sankyo, 10.13039/100004330GlaxoSmithKline (GSK), 10.13039/100005564Gilead, 10.13039/100017981Sysmex, 10.13039/100008067Genomic Health, 10.13039/100018771Veracyte, Sakura, 10.13039/501100018828Leica Biosystems, Lilly, 10.13039/100004319Pfizer, ThermoFisher, Abbvie. Umberto Malapelle has received personal fees (as consultant and/or speaker bureau) from Boehringer Ingelheim, 10.13039/100004337Roche, 10.13039/100030732MSD, 10.13039/100002429Amgen, 10.13039/100011033Thermo Fisher Scientific, Eli Lilly, Diaceutics, GSK, 10.13039/100004334Merck and 10.13039/100004325AstraZeneca, Janssen, Diatech, 10.13039/100004336Novartis and Hedera unrelated to the current work. Umberto Malapelle is Editor in chief of Journal of Liquid Biopsy, Francesco Pepe and Nicola Fusco are member of Editorial Board of the Journal of Liquid Biopsy. No other competing interests to be declared for the remaining authors.

## Figures and Tables

**figs1:**
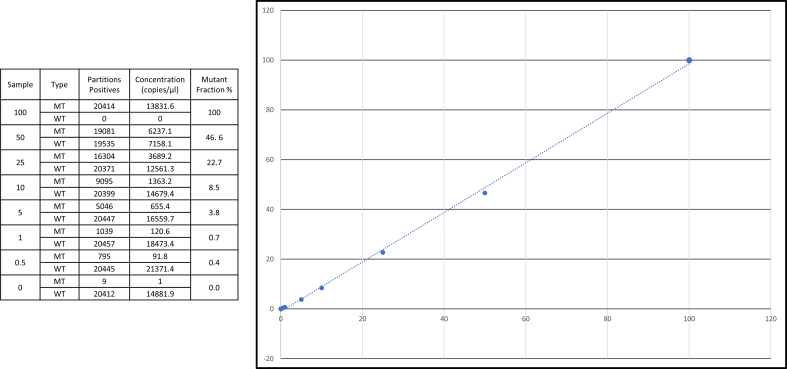


**figs2:**
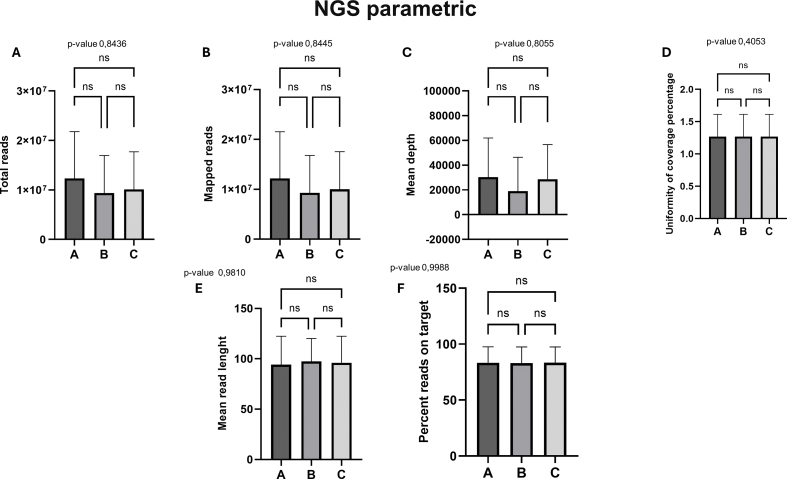


**figs3:**