# Supplementary material for: Harmonization trial on ESR1 testing strategies in ER+/HER2- breast cancer patients: an Italian experience
Source: J Liq Biopsy. 2025 Jul 28;9:100314. doi: 10.1016/j.jlb.2025.100314 (PMC12337205; doi:10.1016/j.jlb.2025.100314)
Supplement: Multimedia component 1 [file mmc1.docx]

|  | **NGS Analysis parameters** | | | | | |
| --- | --- | --- | --- | --- | --- | --- |
|  | **Total reads** | **Mapped reads** | **Mean depth** | **Uniformity of coverage** | **Mean read lenght** | **Percent reads on target** |
| **A** | 3107709 | 3070192 | 10150 | 1.4%* | 106 | 72.5% |
|  | 3229682 | 3194248 | 10434 | 1.4%* | 106 | 71.8% |
| **B** | 1267374 | 1252060 | 4167 | 1.4%* | 105 | 73.7% |
|  | 998930 | 973297 | 3058 | 1.4%* | 101 | 73.1% |
| **C** | 2716286 | 2685371 | 9114 | 1.4%* | 106 | 75.1% |
|  | 984432 | 961445 | 3150 | 1.4%* | 102 | 75.0% |
|  | **dPCR Analysis parameters** | | | | | |
|  | **Copy Variant (cp/ul)** | | **Copy WT (cp/ul)** | | **Variant Partitions** | **WT Partitions** |
| **A** | 62.7 | | 14923.4 | | 547 | 9721 |
|  | 83.2 | | 3897.1 | | 722 | 16653 |
| **B** | 0.7 | | 68.4 | | 6 | 595 |
|  | 0.7 | | 69.8 | | 6 | 608 |
| **C** | 0.1 | | 21.4 | | 1 | 188 |
|  | 0.1 | | 20.3 | | 1 | 178 |

**Supplementary Table 1.** Technical parameters using NGS and dPCR systems from internal validation of standard reference samples A, B, and C by the coordinating institution.
